# Changes in the Blood Viscosity in Patients With SARS-CoV-2 Infection

**DOI:** 10.3389/fmed.2022.876017

**Published:** 2022-06-17

**Authors:** Hayder M. Al-kuraishy, Ali I. Al-Gareeb, Sadiq M. Al-Hamash, Simona Cavalu, Maisra M. El-Bouseary, Fatma I. Sonbol, Gaber El-Saber Batiha

**Affiliations:** ^1^Department of Clinical Pharmacology and Medicine, College of Medicine, Al-Mustansiriya University, Baghdad, Iraq; ^2^Al-Mustansiriya University, Baghdad, Iraq; ^3^Faculty of Medicine and Pharmacy, University of Oradea, Oradea, Romania; ^4^Department of Pharmaceutical Microbiology, Faculty of Pharmacy, Tanta University, Tanta, Egypt; ^5^Department of Pharmacology and Therapeutics, Faculty of Veterinary Medicine, Damanhour University, Damanhour, Egypt

**Keywords:** COVID-19, hyperviscosity syndrome, COVID-19 vaccination, SARS-CoV-2, immunoinflammatory disorders

## Abstract

Coronavirus disease 2019 (COVID-19) is caused by a novel virus known as severe acute respiratory syndrome coronavirus 2 (SARS-CoV-2). SARS-CoV-2-induced hyperinflammation together with alteration of plasma proteins, erythrocyte deformability, and platelet activation, may affect blood viscosity. Thus, this review aimed to study the link between SARS-CoV-2 infection and alteration of blood viscosity in COVID-19 patients. In order to review findings related to hyperviscosity in COVID-19, we suggested a protocol for narrative review of related published COVID-19 articles. Hyperviscosity syndrome is developed in different hematological disorders including multiple myeloma, sickle cell anemia, Waldenstorm macroglobulinemia, polycythemia, and leukemia. In COVID-19, SARS-CoV-2 may affect erythrocyte morphology *via* binding of membrane cluster of differentiation 147 (CD147) receptors, and B and 3 proteins on the erythrocyte membrane. Variations in erythrocyte fragility and deformability with endothelial dysfunction and oxidative stress in SARS-CoV-2 infection may cause hyperviscosity syndrome in COVID-19. Of interest, hyperviscosity syndrome in COVID-19 may cause poor tissue perfusion, peripheral vascular resistance, and thrombosis. Most of the COVID-19 patients with a blood viscosity more than 3.5 cp may develop coagulation disorders. Of interest, hyperviscosity syndrome is more commonly developed in vaccine recipients who had formerly received the COVID-19 vaccine due to higher underlying immunoglobulin concentrations, and only infrequently in those who have not received the COVID-19 vaccine. Taken together, these observations are untimely too early to give a final connotation between COVID-19 vaccination and the risk for development of hyperviscosity syndrome, consequently prospective and retrospective studies are necessary in this regard.

## Introduction

Coronavirus disease 2019 (COVID-19) is a current pandemic disease that began in Wuhan, China in late December 2019. COVID-19 is caused by novel virus known as severe acute respiratory syndrome coronavirus 2 (SARS-CoV-2) which produced a worldwide crisis with high morbidity and mortality (1). It has been shown that COVID-19 led to more than 500 million affected cases with more than 6 million confirmed deaths till late May 2022. Different variants of SARS-CoV-2 strains emerged in the early months of 2020, and the last variant was Omicron SARS-CoV-2, which was mild with moderate transmission and low mortality (2). Up to date, a new variant strain of SARS-CoV-2 named the BA2 subtype has spread in specific regions of China. Besides, a new mutant variant of Omicron SARS-CoV-2 BA1 and BA2 has been observed and detected in the United Kingdom, with about 637 confirmed cases. This new strain has been renamed as the XE variant of SARS-CoV-2, which is now with outstanding spread in China (3). Thus, we are challenged by the emergence of new strains that could be highly virulent and may cause the propagation of new waves.

Most COVID-19 patients are asymptomatic or present with mild flu-like illnesses in about 85% of the cases. However, 15% of COVID-19 patients may present with moderate symptoms, including headache, fever, sweating, arthralgia, myalgia, dry cough, and fatigue (4). However, 5% of COVID-19 patients may develop severe and critical presentations due to the development of acute lung injury (ALI) and acute respiratory distress syndrome (ARDS) (5). COVID-19 patients with ALI/ARDS require ICU admission and mechanical ventilation for respiratory support (6, 7). Moreover, COVID-19 may cause extra-pulmonary manifestations, including neurological complications (8), acute kidney injury (9), testicular injury (10), heart failure (11), new-onset diabetes mellitus (12), and thromboembolic disorders (13).

Of note, SARS-CoV-2 exploits diverse receptor types to reach the affected cells. The angiotensin converting enzyme 2 (ACE2) is an innovator one correlated in the pathogenesis of SARS-CoV-2 infection (14). This interface triggers down-regulation of ACE2, which is essential for alteration of pro-inflammatory/vasoconstrictor angiotensin II (AngII) to vasodilator/anti-inflammatory Ang1-7 (15). Notably, SARS-CoV-2 infection in severe cases may exaggerate human immune responses, leading to hyperinflammation, hypercytokinemia, and cytokine storm (16). Furthermore, SARS-CoV-2-induced hyperinflammation together with alteration of plasma proteins, erythrocyte deformability, and platelet activation may affect blood viscosity (17).

Thus, this narrative review aimed to study the link between SARS-CoV-2 infection and alteration of blood viscosity in COVID-19 patients.

## Method and Search Strategy

In order to review findings related to hyperviscosity in COVID-19, the search was conducted from late December 2019 to early January 2022 by using search engines including MEDLINE, Scopus, Web of Science, PubMed, China National Knowledge Infrastructure, Embase, Wanfang Data, and China Biology Medicine by using the following keywords and terms; COVID-19 or SARS-CoV-2 or 2019-nCov and Hyperviscosity or Erythrocyte deformability or Thrombosis. There were no limitations for language and article types.

## Blood Viscosity and Hyperviscosity Syndrome

Blood viscosity is a measure of blood flow resistance and can also be recognized as the stickiness and thickness of blood (18). The main determinants of blood viscosity are erythrocyte deformability, hematocrit, erythrocyte aggregation, and plasma viscosity, which depend on plasma macromolecules and water content. Hematocrit represents the main determinant of blood viscosity; an increase in hematocrit can elevate it by 4% (19). When the hematocrit rises to 60–70% as in polycythemia, the blood viscosity become higher than water by 10 times with consequent increment resistance to the blood flow. As well, increasing body temperature may induce dehydration with an increase in blood viscosity (20). An increase in blood viscosity leads to the development of hyperviscosity syndrome. Of note, hyperviscosity syndrome is developed in different hematological disorders, including multiple myeloma, sickle cell anemia, Waldenstorm macroglobulinemia, polycythemia, and leukemia (21, 22). Normal BV is usually between 1.4 and 1.8 centipoise (cp), and symptoms of hyperviscosity syndrome develop when blood viscosity exceeds 4.0 cp (23). Patients with hyperviscosity syndrome are presented with diving symptoms due to impairment of blood flow, including headache, confusion, visual disturbances, vertigo, and thrombotic events with or without mucosal hemorrhage (21, 22). Sloop and colleagues found that inflammation and hypergammaglobulinemia together with the fostering of erythrocyte aggregation in sepsis could be the potential mechanisms of increasing blood viscosity in different infectious diseases (24). Hyperviscosity syndrome in severe infections provokes thromboembolic disorders with reduction of tissue perfusion resulting in multi-organ injury (MOI) and fatal outcomes (24).

## Immunological Disorders and Hyperviscosity Syndrome

Blood viscosity is highly sensitive to acute-phase reactants and inflammatory reactions. Thus, acute and chronic inflammatory disorders are linked with elevations of blood viscosity and the development of hyperviscosity syndrome (25). It has been reported that the development of hyperviscosity syndrome was linked with an increase in inflammatory biomarkers like erythrocyte sedimentation rate and C-reactive protein (CRP) (25). Therefore, hyperviscosity syndrome may progress in various immunoinflammatory disorders like rheumatoid arthritis (RA) and systemic lupus erythematosus (SLE) due to formation of intermediate immunocomplex and hyperparaproteinemia respectively (26, 27). Hyperviscosity syndrome in RA patients is correlated with levels of rheumatoid factor, fibrinogen, and inflammatory levels (26). However, hyperviscosity syndrome in RA patients treated with immunosuppressive agents and plasmapheresis is rare (28). Further, hyperviscosity syndrome could be the presenting symptoms in patients with SLE due to the development of monoclonal gammopathy and an unusual increase of immunoglobulin type G4 (29). Moreover, there is an interacted relationship between hyperviscosity syndrome and inflammation due to the increase of acute phase reactant fibrinogen, whose level is correlated with increasing blood viscosity (30). Notably, fibrinogen-related proteins are augmented during the immune response to numerous inflammatory stimuli (31). Fibrinogen and related proteins play a perilous role in neutralizing invading pathogens (31). Sequentially, exaggerated immune responses and exaggerated levels of fibrinogen-related proteins are connected with the development of hyperviscosity syndrome (32).

In addition, abnormal immune response in some viral infections may trigger activation of macrophage cluster of differentiation 169 (CD169), which is involved in immune response and activation of bone marrow for production of erythrocytes (33). Over-activation of CD169 macrophages may be linked with the propagation of polycythemia (33). Besides, CD169 macrophages control immunological responses during viral infections by recruiting monocytes and producing pro-inflammatory cytokines and chemokines (34). In this state, immunological response to various stimuli may increase blood viscosity with the development of hyperviscosity syndrome. These verdicts indicate that abnormal immuno-inflammatory disorders are associated with the progression of hyperviscosity syndrome.

## Viral Infections and Hyperviscosity Syndrome

It has been reported that hyperviscosity syndrome may develop in different viral infections. For example, impaired humoral and cellular immunity may increase immunoglobulin (IgG) levels in patients with human immunodeficiency virus type 1 (HIV-1) infections with subsequent development of hyperviscosity syndrome (35). Increased blood viscosity and the development of hyperviscosity syndrome in HIV-1 infected patients may be related to B cell hyperactivation, increased IgG production, changes in T cell-mediated B cell regulation, chronic exposure to HIV-1 antigens, increased production of interleukin 6 (IL-6), and direct activation of B cells by HIV-1 (36). Likewise, production of myeloma associated IgG1 paraprotein against HIV-1 p24 antigen in HIV-1 patients (37).

Moreover, indicators of blood viscosity are augmented in patients with hepatitis B virus (HBV) infection (38). A prospective study revealed that patients with HBV infection had greater RBCs aggregation index, hematocrit, and blood viscosity as compared with control groups (38). As well, soluble fibrinogen like protein 2 (sFGL2) is elevated in patients with HBV infection (39). Into the bargain, hyperviscosity syndrome has been reported to be linked with respiratory viral infections like influenza pneumonia (40). In their study, Bogomolov et al. observed that influenza pneumonia and other severe acute respiratory viral infections can cause hyperviscosity syndrome through induction of hypercoagulation, alteration of fibrinolytic activity, intravascular homeostasis, and failure of microcirculation (40). High blood viscosity in influenza pneumonia and respiratory viral infections may provoke progression of thrombosis due to an increase in vascular resistance, which hampers peripheral tissue perfusion (24). Piñol-Ripoll and coworkers found that chronic bronchitis predisposes to the development of hyperviscosity syndrome and an increased risk of ischemic stroke (41). Thus, these observations point out that acute respiratory viral infections as well as other viral infections may increase the risk of development of vascular complications through induction and progression of viral infections.

## COVID-19 and Hyperviscosity Syndrome

SARS-CoV-2 infection has been shown to reduce erythrocyte deformability and increase erythrocyte aggregation in COVID-19 patients in low-shear flow and stasis, which, combined with an increase in fibrinogen level, may increase blood viscosity and lead to the development of hyperviscosity syndrome (42). Increasing blood viscosity and hyperviscosity syndrome progression in COVID-19 may be linked to a variety of mechanisms, including endothelial dysfunction, exaggerated immune response, hypoxia, and coagulation disorders (17). Likewise, platelet hyper-reactivity, high ferritin, and P-selectin activity together with changes in erythrocyte function in COVID-19 might participate in the development of hyperviscosity syndrome (43). In severe SARS-CoV-2 infections, fever and dehydration due to anorexia, vomiting, and diarrhea may increase blood viscosity in COVID-19 patients (44).

Concerning the clinical perspective regarding the potential role of SARS-CoV-2 infection in the propagation of hyperviscosity syndrome, SARS-CoV-2 infection is linked with microcirculation failure in hospitalized COVID-19 patients (42). Of note, microcirculatory failure in COVID-19 patients leads to noteworthy alterations in the erythrocytes deformability and aggregation, resulting in stasis and augmentation of blood viscosity (45). Besides, coagulation disorders, endothelial dysfunction, and cytokine storm all contribute to microcirculation dysfunction in septic COVID-19 patients (46). The Renoux et al. study, which included seven hospitalized COVID-19 patients, seven non-COVID-19 septic patients, and seven healthy controls, found that erythrocyte deformability was lower in both COVID-19 patients and non-COVID-19 septic patients compared to controls (42). In addition, erythrocyte aggregation was higher in COVID-19 patients as compared to non-COVID-19 patients without noteworthy variations in fibrinogen levels and blood viscosity (42). This small sample size study may not give a tangible clue regarding normal blood viscosity in COVID-19. However, a retrospective study including 41 COVID-19 patients reported that assessed blood viscosity was superior in COVID-19 patients compared with healthy control subjects (17).

### Hyperviscosity Syndrome and Inflammatory Signaling Pathways in COVID-19

Exaggerated immune response and the release of pro-inflammatory cytokines, primarily IL-6, have been linked to the development of cytokine storm and MOI (47). In COVID-19, IL-6 is thought to be an important activator of fibrinogen synthesis (48). In addition, deregulation of the renin-angiotensin system (RAS) with an increase in circulating AngII levels in COVID-19 may prompt expression and synthesis of fibrinogen (49). In turn, high fibrinogen levels activate erythrocyte membrane integrinαvβ3 receptors, which induce erythrocyte aggregation and the development of hyperviscosity syndrome (48). Of interest, CD169 macrophages, which are involved in the maturation of erythrocytes, are activated in SARS-CoV-2 infection, resulting in polycythemia and the development of hyperviscosity syndrome (50). It has been observed that CD169 monocytes are expressed in 93.7% of COVID-19 patients and are regarded as having diagnostic benefits (50). Consequently, SARS-CoV-2-induced expression of CD169 by macrophages/monocytes may promote the development of polycythemia and hyperviscosity syndrome in COVID-19.

Significantly, increased blood viscosity in COVID-19 patients stimulates the release of arginine vasopressin (51), which causes the release of pro-inflammatory cytokines *via* activation of the nuclear factor kappa B (NF-κB) and nod-like receptor pyrin 3 (NLRP3) inflammasomes, both of which contribute to increased blood viscosity (51). Of note, both of NF-κB and NLRP3 inflammasome persuade asymmetry of erythrocyte membrane with decrease of erythrocyte deformability in normal and sickle erythrocytes (52, 53). Besides, NF-κB and NLRP3 inflammasome are extremely triggered in COVID-19 (54), and might a latent causes for lessening of erythrocyte deformability in COVID-19.

Moreover, p38 mitogen activated protein kinase (p38MAPK), mechanistic target of rapamycin (mTOR) and high mobility group box protein 1 (HMGP1) are also activated in COVID-19, leading to the release of pro-inflammatory cytokines (55–57). In turn, increased pro-inflammatory cytokines promote elevation of blood viscosity by inducing expression of fibrinogen with a reduction of erythrocyte deformability (58). Likewise, COVID-19 is usually associated with psychological stress and sympathetic outflow (59). In relevant, psychological stress increases circulating AngII as well, AngII promotes psychological stress through augmentation of sympathetic activation (60). Similarly, AngII receptor blockers attenuate stress pressor in young adults (60). Therefore, COVID-19-induced psychological stress may augment the dysregulated RAS by increasing AngII with the consequent development of hyperviscosity syndrome. As well, high circulating AngII in COVID-19 promotes the release of pro-inflammatory cytokines with the induction of erythrocyte aggregation and an increase in blood viscosity (61).

These observations suggest that activated inflammatory signaling pathways and the release of pro-inflammatory cytokines might be the latent causes for the development of hyperviscosity syndrome in COVID-19.

### Hyperviscosity Syndrome and Erythrocyte Deformability in COVID-19

In COVID-19, SARS-CoV-2 may affect erythrocyte morphology *via* binding of membrane cluster of differentiation 147 (CD147) receptors and Band3 protein on the erythrocyte membrane (62, 63). These changes reduce the functional capacity of erythrocytes for oxygen transport and result in the development of tissue hypoxia (63). It has been shown that erythrocyte distribution width and other indices were brutally affected in SARS-CoV-2 infection and were associated with COVID-19 severity (64). Besides, severe hypoxia and acidosis encourage changes in the erythrocyte morphology (65). These explanations propose that direct SARS-CoV-2-induced erythrocyte dysmorphology and connected metabolic acidosis with hypoxia may induce the development of hyperviscosity syndrome in COVID-19.

Moreover, lipoproteins can disturb blood viscosity as low density lipoprotein (LDL) is clearly correlated while high density lipoprotein (HDL) is negatively correlated with blood viscosity (66). Indeed, HDL is required for erythrocyte morphology and deformability; thus, a decrease in HDL may shorten erythrocyte life by increasing osmotic fragility and decreasing erythrocyte deformability (67). In COVID-19, there is a notable variation in lipoprotein serum levels, and low HDL levels are linked with COVID-19 severity (68, 69). Thus, the decrease of HDL in SARS-CoV-2 infection may increase blood viscosity with the development of hyperviscosity syndrome in COVID-19.

Notably, COVID-19-induced oxidative stress may prompt an increase in blood viscosity (70). High oxidative stress in COVID-19 can trigger atypical hemorheological alterations with a decrease in erythrocyte deformability (71). In severe SARS-CoV-2 infections, oxidative stress may lead to endothelial dysfunction and thrombotic complications (72). Hence, variations in erythrocyte fragility and deformability with endothelial dysfunction and oxidative stress in SARS-CoV-2 infection may cause hyperviscosity syndrome in COVID-19.

Remarkably, erythrocyte morphology and functions are also affected in SARS-CoV-2 infection with the progression of erythrocrine dysfunction (73). In this state, the development of abnormal erythrocytes may contribute to the development of endothelial dysfunction and vascular injury by aggregate oxidative stress (74). Of interest, erythrocytes from COVID-19 patients promote expression of endothelial arginase with the generation of reactive oxygen species (ROS), reduction of endothelial NO and development of endothelial dysfunction (74). Thus, SARS-CoV-2 infection-induced oxidative stress might in part be mediated by the development of abnormal erythrocytes in COVID-19.

### Hyperviscosity Syndrome and Thrombosis in COVID-19

Conspicuously, severe COVID-19 is linked with the development of thromboembolic events due to direct SARS-CoV-2 cytopathic effects and related platelet activation, coagulation activation, endothelial dysfunction, and inhibition of the fibrinolytic pathway (75). Also, down-regulation of ACE2 with deregulation of RAS together with exaggerated release of pro-inflammatory cytokines may induce endothelial dysfunction through reduction of prostacyclin and nitric oxide (NO) (76). Thrombotic events may increase the risk of the development of hyperviscosity syndrome (77). These observations suggest a mutual interaction between HVS and thrombotic events in COVID-19.

Additionally, hypoalbuminemia is linked with an increase in blood viscosity and the development of hyperviscosity syndrome (78). Of note, serum albumin is negatively correlated with D-dimer and CRP, and hypoalbuminemia is linked with the development of coagulopathy in COVID-19 patients through a decrease in the anticoagulant and antiplatelet effects of albumin (79). A study of 113 COVID-19 patients by Bi et al. found that a high fibrinogen/albumin ratio was associated with an increased risk of thrombotic events, disease severity, and poor clinical outcomes (80). Thus, the blood viscosity is increased and reaches up to 4.2 cp. Consequently, hyperfibrinogenemia and hypoalbuminemia may increase blood viscosity and contribute to the progression of hyperviscosity syndrome and thrombotic complications in COVID-19 (80).

Strangely, most of the COVID-19 patients with higher blood viscosities of more than 3.5 cp may develop coagulation disorders (81). In this condition, there is a close relationship between hyperviscosity syndrome and thrombotic events in COVID-19. It has been shown that critical COVID-19 patients were associated with thrombotic complications and blood viscosity greater than 3.5 cp (the normal range is 1.4–1.8 cp) was correlated with thrombotic complications (81). In addition, Truong et al. reported that symptoms of hyperviscosity syndrome were more obvious in COVID-19 patients with a blood viscosity of more than 4.2 cp (82). These findings suggest that higher blood viscosity is connected with more severe hyperviscosity syndrome in COVID-19.

**FIGURE 1:**
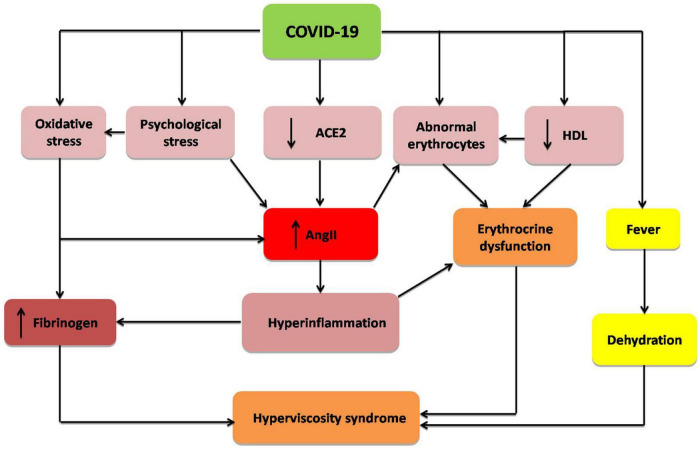
Mechanism of hyperviscosity syndrome in COVID-19: COVID-19 through down-regulation of angiotensin converting enzyme 2 (ACE2), psychological stress, hyperinflammation, oxidative stress, abnormal morphology of erythrocytes, and reduction of high density lipoprotein (HDL). These changes increase fibrinogen level and angiotensin II (AngII), with induction of erythrocrine dysfunction and subsequent development of hyperviscosity syndrome.

These verdicts propose that severe SARS-CoV-2 infection in COVID-19 patients can increase blood viscosity by modulating fibrinogen, albumin, lipoproteins, and erythrocyte deformability and aggregations (Figure 1).

### Complications of Hyperviscosity Syndrome in COVID-19

Of interest, hyperviscosity syndrome in COVID-19 may cause poor tissue perfusion, peripheral vascular resistance, and thrombosis (24). In particular, low-shear areas are vulnerable to thrombosis due to a decrease in the dispersion of clotting factors and a reduction in the shear-induced release of antithrombotic molecules like NO and prostacyclin (24).

Indeed, hyperviscosity syndrome may lead to extra-pulmonary complications, including acute kidney injury, skeletal muscle ischemia, glucose intolerance, and myocardial necrosis (83). In addition, hyperviscosity syndrome leads to ventilation-perfusion mismatch and the development of pulmonary hypoperfusion. These pathological changes lead to silent hypoxemia and exaggerated pulmonary vascular resistance (84). Furthermore, COVID-19-induced hyperviscosity syndrome has been associated with numerous cardiovascular and neurological complications like stroke and myocardial infarction (85, 86). In particular, hyperviscosity syndrome increases the risk of the development of myocardial infarction in COVID-19 patients (87). As well, immunothrombosis and endothelial dysfunction, which are induced by SARS-CoV-2 infection, could be potential causes of hyperviscosity syndrome in COVID-19 (82). These vicissitudes escalate the risk of the development of myocardial infarction in surviving COVID-19 patients due to the progression of coronary microangiopathy (88).

**FIGURE 2:**
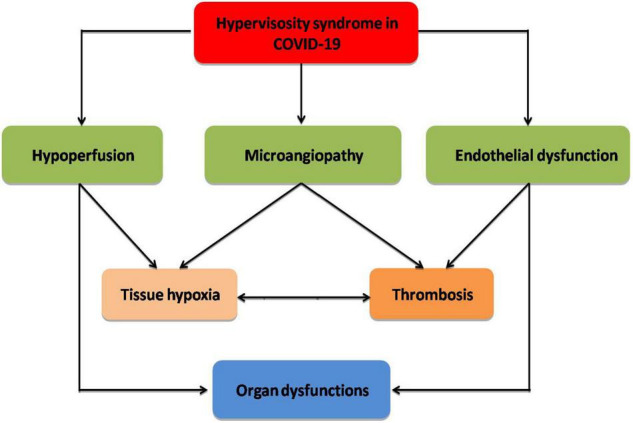
Complications of hyperviscosity syndrome in COVID-19: Hyperviscosity syndrome provokes the development of endothelial dysfunction, microangiopathy, and hypoperfusion with the development of thrombosis and tissue hypoxia, which eventually cause organ dysfunction.

Indeed, hyperviscosity syndrome is connected with the progression of post-COVID-19 syndrome (long COVID-19), which is characterized by dyspnea, fatigue, cognitive dysfunction, and headache following recovery from COVID-19 (89). It has been shown that long COVID-19 is linked with cardio-pulmonary fibrosis and immunosuppression due to upregulation of transforming growth factor beta (90). Protracted inflammatory changes and high blood viscosity in patients with long COVID-19 can decrease tissue perfusion with induction of abnormal cellular metabolism (91). In this state, COVID-19-induced abnormal erythrocrine function may promote tissue hypoxia and subnormal cell metabolism, which may prolong symptoms of long COVID-19 (74). Herein, hyperviscosity syndrome with or without erythrocrine dysfunction in COVID-19 contributes to the decrease in tissue oxygenation and the development of cardio-metabolic complications in long COVID-19 (Figure 2).

## COVID-19 Vaccination and Hyperviscosity Syndrome

The management of COVID-19 heavily relies on the presence of safe and effective vaccines. There are various types of vaccines against SARS-CoV-2. One type is mRNA vaccines encoding the S protein antigen of the virus, like the Pfizer-BioNTech COVID-19 Vaccine (92). Another type of COVID-19 vaccine is the vector-based vaccine that delivers the code for the spike antigen of SARS-CoV-2. Examples of vector-based vaccines include the Oxford–AstraZeneca vaccine, Convidecia vaccine, Sputnik-V vaccine, and Johnson vaccine (93). Also, there are inactivated vaccines, such as the Sinopharm vaccine (93). Another potential COVID-19 vaccine is the NVX-CoV2373 vaccine, which contains a recombinant nanoparticle spike protein (94). The COVID-19 vaccine was developed in the early part of April 2020 to control the spread of the SARS-CoV-2 infection (95). It is of note that the FDA approved the first COVID-19 vaccine on August 23, 2021, which is an mRNA vaccine that has been known as the Pfizer-BioNTech COVID-19 Vaccine. This vaccine was approved for those who are 16 years of age or older (95). Subsequent to the COVID-19 vaccination, some reports disclosed that the blood viscosity was augmented due to induction of immune response and an increase in anti-SARS-CoV-2 immunoglobulins (96). It has been shown that hyperviscosity syndrome may develop following COVID-19 vaccination, causing immunoinflammatory changes (96). Hyperviscosity syndrome is associated with the concentration of immunoglobulins; nevertheless the lowest normal immunoglobulins concentrations are below 545 mg/dl whereas the lowest blood viscosity is 1.5 cp (97). The blood viscosity will be 2.6 cp when the immunoglobulin concentrations reach up to 6160 mg/dl (94). Of note, symptoms of HVS develop when BV exceeds 4.0 cp (97).

Normally, in healthy COVID-19 vaccine recipients, the blood viscosity is increased by 2.4 cp (98). However, COVID-19 vaccine-induced hyperviscosity syndrome is more common in patients with metabolic syndrome due to metabolic disorders which increase blood viscosity (99). Of interest, hyperbilirubemia in chronic liver diseases may induce the development of hyperviscosity syndrome following COVID-19 vaccination (99). Interestingly, hyperbilirubinemia provokes the development of hyperviscosity syndrome by an unknown mechanism (99). Therefore, patients with metabolic disorders are regarded as high-risk factors for the development of hyperviscosity syndrome after COVID-19 vaccination. Hence, monitoring of blood viscosity in COVID-19 vaccine recipients is compulsory to avoid post-vaccine complications (100, 101).

It has been reported that patients with metabolic syndrome had higher blood viscosity and were more susceptible to the propagation of hyperviscosity syndrome (102). In particular, metabolic syndrome is associated with underlying systemic inflammation and oxidative stress, which increases the blood viscosity by reducing erythrocyte deformability (103). Consequently, patients with metabolic syndrome are at a superior risk for the development of hyperviscosity syndrome following COVID-19 vaccination. Herein, COVID-19 vaccinations may increase the risk for development of hyperviscosity syndrome in patients with metabolic syndrome (104). It has been demonstrated that the blood viscosity was elevated by 2.7 times in healthy subjects compared to 2.99 times in patients with metabolic syndrome after COVID-19 vaccinations (104). This elevation in the blood viscosity did not reach the state of hyperviscosity syndrome, which might be due to the validity of the method in the assessment of blood viscosity (105).

Remarkably, oxidative stress can persuade a reduction in erythrocyte deformability with a successful increase in blood viscosity (106). High oxidative stress and fibrinogen together with prolonged low-grade inflammation in obesity are related to the development of hyperviscosity syndrome (107, 108). Thus, obese patients are at great risk for the development of hyperviscosity syndrome following COVID-19 vaccination. Likewise, the immune response in obese patients to the COVID-19 vaccine is weak due to the decreased reactivity of lymphocytes (109). Hence, interruption of the immune response may reduce the concentration of immunoglobulins after COVID-19 vaccination (110). As well, the immune response in obese patients was low after the influenza vaccine (110).

Astonishingly, hyperviscosity syndrome is more commonly developed in vaccine recipients who have formerly received the COVID-19 vaccine due to higher underlying immunoglobulin concentrations and only infrequently in those who have not received the COVID-19 vaccine (96). Therefore, screening of subjects for previous COVID-19 vaccination is vital before introducing COVID-19 vaccination to avert the development of hyperviscosity syndrome and related complications. Besides, use of contraceptives may increase the risk of development of hyperviscosity syndrome following COVID-19 vaccination (111). Hence, we suggest taking the risk into consideration for patients taking contraceptives at the time of COVID-19 vaccination.

Taken together, these findings are too preliminary to draw any conclusions about the relationship between COVID-19 vaccination and the risk of developing hyperviscosity syndrome; therefore, further research, both prospective and retrospective, is required.

The present review had numerous limitations, including the scarcity of prospective studies which appraised the blood viscosity of COVID-19. As well, most of the studies were hypothetical in their explanation of hyperviscosity syndrome in COVID-19 and COVID-19 vaccination. However, regardless of these limitations, the present critical review reveals that hyperviscosity syndrome is an imperative mechanistic pathway in the progression of COVID-19 complications and associated vaccines.

## Conclusion

The present review showed that COVID-19 and linked vaccines are associated with the development of hyperviscosity syndrome, particularly in patients with previous COVID-19 and metabolic disorders. The potential mechanism of hyperviscosity syndrome in COVID-19 and COVID-19 vaccines is augmentation in the levels of fibrinogen and immunoglobulins. As well, dehydration, oxidative stress, and inflammatory reactions could be additional contributing factors in the development of hyperviscosity syndrome in COVID-19. Though, this review did not determine the ultimate causal relationship between COVID-19 and COVID-19 vaccines with the development of hyperviscosity syndrome. Therefore, experimental, *in vitro*, and clinical studies are necessary in this regard.

## Author Contributions

HA-k and AA-G performed data collection and analysis. HA-k, AA-G, SC, SA-H, ME-B, FS, and GE-SB wrote the first draft of the manuscript and all authors commented on previous versions of the manuscript. All authors contributed to the study conception and design and read and approved the final manuscript.

## Conflict of Interest

The authors declare that the research was conducted in the absence of any commercial or financial relationships that could be construed as a potential conflict of interest.

## Publisher’s Note

All claims expressed in this article are solely those of the authors and do not necessarily represent those of their affiliated organizations, or those of the publisher, the editors and the reviewers. Any product that may be evaluated in this article, or claim that may be made by its manufacturer, is not guaranteed or endorsed by the publisher.
